# The future of artificial intelligence in thoracic surgery for non-small cell lung cancer treatment a narrative review

**DOI:** 10.3389/fonc.2024.1347464

**Published:** 2024-02-13

**Authors:** Namariq Abbaker, Fabrizio Minervini, Angelo Guttadauro, Piergiorgio Solli, Ugo Cioffi, Marco Scarci

**Affiliations:** ^1^ Division of Thoracic Surgery, Imperial College NHS Healthcare Trust and National Heart and Lung Institute, London, United Kingdom; ^2^ Division of Thoracic Surgery, Luzerner Kantonsspital, Lucern, Switzerland; ^3^ Division of Surgery, Università Milano-Bicocca and Istituti Clinici Zucchi, Monza, Italy; ^4^ Division of Thoracic Surgery, Policlinico S. Orsola-Malpighi, Bologna, Italy; ^5^ Department of Surgery, University of Milan, Milan, Italy

**Keywords:** NSCLC, artificial intelligence, thoracic surgery, deep learning - artificial intelligence, lung cancer

## Abstract

**Objectives:**

To present a comprehensive review of the current state of artificial intelligence (AI) applications in lung cancer management, spanning the preoperative, intraoperative, and postoperative phases.

**Methods:**

A review of the literature was conducted using PubMed, EMBASE and Cochrane, including relevant studies between 2002 and 2023 to identify the latest research on artificial intelligence and lung cancer.

**Conclusion:**

While AI holds promise in managing lung cancer, challenges exist. In the preoperative phase, AI can improve diagnostics and predict biomarkers, particularly in cases with limited biopsy materials. During surgery, AI provides real-time guidance. Postoperatively, AI assists in pathology assessment and predictive modeling. Challenges include interpretability issues, training limitations affecting model use and AI’s ineffectiveness beyond classification. Overfitting and global generalization, along with high computational costs and ethical frameworks, pose hurdles. Addressing these challenges requires a careful approach, considering ethical, technical, and regulatory factors. Rigorous analysis, external validation, and a robust regulatory framework are crucial for responsible AI implementation in lung surgery, reflecting the evolving synergy between human expertise and technology.

## 1 Background

The complexity of lung cancer, characterized by diverse histological subtypes, molecular variations, and intricate staging, necessitates a nuanced approach to diagnosis, treatment, and postoperative surveillance. Traditionally, these challenges have relied heavily on the expertise of pathologists, surgeons, and oncologists. However, the advent of AI has introduced a paradigm shift in how we comprehend, diagnose, and treat lung cancer.

Artificial intelligence (AI) has emerged as a transformative force in many industries (1–3). Mainly due to its spectrum of approaches that range from those striving to replicate human reasoning for effective problem-solving, to others that bypass human reasoning entirely and rely solely on extensive datasets to formulate a framework for addressing specific questions of interest (4, 5). AI technologies In thoracic surgery, primarily through machine learning (ML) techniques and natural language processing (NLP), have shown remarkable potential in enhancing the accuracy of diagnoses, the efficiency of treatments, and the effectiveness of postoperative care. ML algorithms are increasingly being utilized for detailed analysis of imaging data, aiding in the detection and classification of lung cancer, while NLP is transforming the way clinical data and patient histories are processed and interpreted (3). Thus, facilitating better clinical decision-making, minimizing medical errors, and elevating the overall quality and efficiency of patient care (2, 3).

However, the application of AI in thoracic surgery is not without its challenges. Issues such as data privacy, potential biases in AI algorithms, ethical concerns and the need for large, well-annotated datasets for training are significant concerns that need addressing. Moreover, there is a delicate balance between the benefits of AI-assisted decision-making and the preservation of the critical role of medical professionals in patient care (6). This narrative review explores the current state of AI integration in thoracic surgery for non-small cell lung cancer treatment. The subsequent sections provide a detailed examination of AI’s role in preoperative planning, intraoperative guidance, and postoperative management, offering a comprehensive overview of its potential benefits and ongoing challenges.

## 2 Method

A review of the literature was conducted using PubMed, EMBASE and Cochrane to identify the latest research on artificial intelligence and lung cancer which is used to generate a narrative review.

## 3 Pre-operative planning

### 3.1 AI in diagnostics

Lung cancer, a leading cause of cancer-related mortality, necessitates precise histopathological diagnosis for effective therapeutic strategies. Molecular-targeted therapy and immunotherapy advancements have improved outcomes, but classifying subtypes, especially in poorly differentiated carcinomas, remains challenging.

To address these challenges, computational pathology, which involves the use of advanced technologies such as Whole Slide Images (WSI) and deep learning methods, has emerged as a promising avenue. One notable study by Kanavati et al. (7) focused on utilizing a convolutional neural network (CNN) and recurrent neural network (RNN) to predict subtypes of lung carcinoma, with a specific emphasis on transbronchial lung biopsies (TBLB). The deep learning model was meticulously trained on a substantial number of WSIs, prioritizing cases with poor differentiation. The study explored two distinct approaches for WSI diagnosis, both of which demonstrated exceptional accuracy in classifying adenocarcinoma (ADC), squamous cell carcinoma (SCC), and small cell lung carcinoma (SCLC) across diverse datasets.

In situations where diagnostic efficacy is hindered by sparse biopsy materials, AI becomes a pivotal tool, offering guidance to pathologists. Despite challenges posed by limited datasets in cytological slides, studies such as those conducted by Teramoto et al. (8) and Tsukamoto et al. (9), recognize the potential of deep convolutional neural networks (DCNNs) in the classification of these challenging microscopic images. These studies report promising accuracy rates in categorizing lung cancer cells, comparable to human cytotechnologists or pathologists.

Moreover, AI’s significance becomes apparent in scenarios requiring special immunohistochemical staining for differential diagnosis. Studies by Baxi et al. (10) and Wang et al. (11) demonstrate how AI provides high-accuracy guidance from haematoxylin and eosin (H&E)-stained slides unaided by supplementary staining, reducing diagnostic subjectivity. A reduction is critical in the preoperative period, enhancing the reliability of diagnostic conclusions and, consequently, treatment decisions preoperatively.

Collectively, these advancements underscore the evolving landscape of AI applications in pathology, particularly in lung cancer diagnosis, and emphasize the potential for further research to refine classification methodologies and achieve comprehensive cell and array categorization.

### 3.2 Prediction of molecular biomarkers

With personalized cancer treatment, the integration of artificial intelligence (AI) with molecular biology presents a promising avenue, transcending conventional histopathology. This is exemplified in the challenges associated with distinguishing between adenocarcinoma (ADC) and squamous cell carcinoma (SCC) based on a single H&E slide obtained from a small biopsy or cytological material. To ensure precision in diagnosis, supplementary staining for immunohistochemical biomarkers such as TTF-1, CK5/6, CK7, pan keratin, p40, p63 and histochemical stains such as periodic acid-Schiff - PAS becomes essential. Numerous studies have tackled binary classification issues related to subtyping non-small cell lung cancer (NSCLC) from H&E slides, aiming for accurate and swift diagnoses. These studies predominantly feature ADC and SCC Whole Slide Images (WSIs), often sourced from The Cancer Genome Atlas dataset (12–15).

For example, Chen et al. (16) and Pao et al. (17) demonstrated the proficiency of DL in predicting ALK rearrangements and EGFR mutations, respectively, achieving commendable Area Under the Curve (AUC) values. These findings lay a foundation for personalized treatment strategies based on the molecular characteristics of the tumor. Moreover, Chen et al. (16) introduced WIFPS, a deep learning system capable of predicting lung cancer-related immunohistochemistry (IHC) phenotypes directly from H&E histopathologic slides. WIFPS exhibits high consistency with pathologists, offering potential assistance in accurate cancer subtyping, especially in scenarios where traditional methods are unavailable. While WIFPS shows promise in reducing the diagnostic ambiguity of cases labeled as “not otherwise specified” (NOS) and guiding targeted therapy, caution is exercised regarding the necessity for extensive validation studies and the translation of clinical benefits.

In the preoperative phase, the predictive capabilities of molecular biomarkers through AI offer valuable insights into the tumor’s molecular landscape. This foresight enables the tailoring of treatment strategies based on the specific molecular characteristics of the tumor offering a personalized approach to optimizing therapeutic outcomes even in situations of biopsy scarcity. A factor that could lead in future to even less invasive approaches to cancer sampling. The adept handling of the intricacies of molecular data by AI adds a layer of sophistication to the preoperative decision-making process, which could ensure precision in aligning interventions with the unique molecular profile of the tumor.

### 3.3 Imaging and staging

In the preoperative evaluation of lung cancer, radiological imaging plays a pivotal role in further guiding clinical decisions related to staging and subsequent therapeutic pathways. AI applications in this are designed to enhance precision in tumor staging and prognostic assessments, integrating algorithms with established imaging modalities (18). This synergy, especially evident in CT image analysis, can result in a noticeable refinement of tumor staging methodologies (19).

The evaluation of AI-assisted CT diagnostic technology for classifying pulmonary nodules, as delineated by Huang et al. (20) study, showcases remarkable diagnostic performance with an exemplary Area Under the Curve (AUC) of 0.95, complemented by sensitivity, specificity, Positive Likelihood Ratio (PLR), and Negative Likelihood Ratio (NLR) values of 0.90, 0.89, 7.95, and 0.11, respectively. Such significant diagnostic prowess emphasizes the potential impact on lung cancer detection. From the physician’s perspective, the study reported that their perception indicates widespread adoption in tertiary hospitals, citing reduced workload and enhanced efficiency. However, concerns about diagnostic accuracy, misdiagnosis risks, and patient privacy temper enthusiasm.

A broader systematic review of 14 studies by Amir et al. (19) reinforces the efficacy of AI-assisted diagnostic technology in the context of lung cancer. Employing observer-performance studies and Receiver Operating Characteristic (ROC) analyses, the review identified significant accuracy improvement in eight out of nine instances, affirming the beneficial impact of Computer-Aided Diagnosis (CADx) on lung cancer assessments. Despite variations in algorithm categories and a need for further data, the review supports the conclusion that CADx is poised for broader lung cancer screening and holds implications for advancing medical diagnostics across diverse organ systems, aligning with the evolving landscape of non-radiologic screening modalities. Limitations, including potential biases and a focus on Chinese public tertiary hospitals, warrant consideration in interpreting the generalizability of these findings.

In essence, AI’s role in the preoperative period could be transformative for not only refining diagnostic accuracy in histopathology and predicting molecular biomarkers but also augmenting the precision of imaging-based staging, prognostic and screening assessments.

### 3.4 Surgical candidacy

In preoperative planning, surgical candidacy is usually a complex decision involving scientific, ethical, and legal aspects, especially in patients with pre-existing respiratory and cardiovascular conditions. Traditional risk indices, such as Goldman index for cardiac risk, and Torrington index for respiratory risk, while effective in classifying patients into risk groups, lack specificity and sensitivity for individualized operative risk assessment (21). The identification of lung nodules and their classification using AI has been shown to be superior to human identification in experimental studies. Esteva H, et al. (21)looked at comparing artificial Neural Networks (NN), which were designed to emulate the human neural system to estimate the postoperative prognosis comparatively to traditional risk indices following lung resection. NN was found to offer a more flexible and individualized approach with nearly 100% sensitivity and specificity for predicting patient outcomes. Similarly, Santos-Garcia G, et al. (22) found similar outcomes when using artificial NN, which offered high performance in predicting postoperative cardio-respiratory morbidity.

In lung cancer surgery, conventional methods, such as video-assisted thoracic surgery (VATS), have demonstrated benefits in terms of reduced trauma, faster recovery, and fewer complications. However, limitations exist, including blind spots in the operation and constraints on the flexibility of surgical instruments (23). AI promises to revolutionize surgical practices by providing real-time analysis of intraoperative progress, enhancing decision-making capabilities, and ultimately improving surgical outcomes.

The studies by Chang et al. (24) and Etienne et al. (25) both underscore the transformative role of artificial intelligence (AI) in reshaping medical practices, albeit in distinct domains. Chang et al. (24) delved into pre-anesthetic consultations, emphasizing the pervasive trend of comprehensive digitalization in recent medical practices. Their study illuminates the potential of AI to harness historical medical data for accurate predictions, avoiding invasive interventions. The presented AI-assisted prediction model not only facilitates integrated risk assessments but also addresses the challenges of dynamic data adjustments through manual input by clinicians, ensuring adaptability to diverse patient records.

In contrast, Etienne et al. (25) focused on thoracic surgery, particularly lobectomies and pneumonectomies for non-small cell lung cancer. Their exploration of AI as a decision-making aid in surgical risk assessment and prognosis aligns with Change et al.’s emphasis on individualized medicine. Both studies acknowledge the limitations of traditional risk indexes and highlight the precision and adaptability offered by AI in evaluating individual risk factors.

Etienne et al. (25) cite studies by Santos-Garcia et al. (22) and Esteva et al. (21), showcasing AI’s successful application in predicting cardio-respiratory morbidity and post-operative prognosis for nonsmall cell lung cancer. This aligns with Chang et al. (24) proposition that AI, specifically the Naïve Bayes Classifier, is an optimal tool for predictive modeling. Furthermore, both studies stress the potential of AI collaborations among medical specialists, with applications ranging from distinguishing lung adenocarcinoma and squamous cell carcinoma to outperforming pulmonologists in interpreting pulmonary function tests.

Despite the promising outlook, both studies acknowledge challenges for broader AI acceptance. Chang et al. (24) discuss the drawbacks of traditional mathematical equation-based approaches, contrasting them with the adaptability and real-time capabilities of AI. Etienne et al. (25) specifically highlight the need for AI to address complex clinical questions, especially those involving patient comorbidities, to fully integrate into clinical practice.

In synthesis, these studies collectively show the potentially pivotal role of AI in reshaping medical decision-making, whether in pre-anaesthetic consultations or thoracic surgery. The emphasis on individualized, adaptive approaches and collaboration among medical specialists reflects a shared vision of AI as a transformative force in the future of medicine.

## 4 Intraoperative period

### 4.1 Surgical guidance and precision

The integration of artificial intelligence (AI) technologies is reshaping the intraoperative landscape of lung cancer management, notably in surgical precision and guidance. This is exemplified by Kanavati et al. (26) study which highlights the pivotal role of deep learning (DL) methodologies in real-time guidance. These DL algorithms, honed on extensive datasets, exhibit unparalleled precision in outlining tumor boundaries intraoperatively, surpassing human visual capabilities (26). This precision translates into tangible benefits during surgery, minimizing the risk of inadvertent tissue damage and optimizing tumor resection (27). The fusion of AI with intraoperative imaging modalities, showcased by Kanavati et al. (26) underscores the potential for informed decision-making, elevating the intraoperative environment’s dynamic nature (26, 27).

Intraoperative imaging has evolved from X-rays to technologies like C-arm, intraoperative ultrasound (US), and intraoperative MRI. Molecular imaging, especially in radio-guided surgery, utilizes tracers like radioactive, fluorescent, magnetic, or hybrid options. Emerging technologies like multispectral optoacoustic tomography (MSOT), fiber-based microscopy, and Raman spectrometry contribute to these advancements.

The integration of AI with intraoperative imaging, exemplified by studies such as Kanavati et al. (21), is reshaping lung cancer surgery. DL methods, trained on extensive datasets, exhibit remarkable precision in real-time guidance, exceeding human capabilities. This convergence enhances surgical precision, minimizing the risk of unintended tissue damage, and optimizing tumor removal. Combining AI with intraoperative imaging not only aids decision-making but also injects dynamism into the surgical environment.

Navigation and visualization concepts for preoperative images seamlessly extend to intraoperative molecular images. Technologies like freehand SPECT, incorporating augmented reality (AR) and pointer navigation, exemplify this integration (28) The adaptability of intraoperative imaging to tissue changes, even post-lesion removal, and real-time feedback from methods like radio- or fluorescence guidance confirm successful lesion localization. This fusion of AI-guided precision and advanced intraoperative imaging transforms surgical practices and elevates the field’s decision-making capabilities (28, 29).

### 4.2 Augmented reality and navigational assistance

The landscape of thoracic surgery could be undergoing a significant shift merging technology and precision. Li et al.’s (29) exploration dive into the long-standing use of thoracoscopic lobectomy for lung cancer, challenged by the rise in small tumor discoveries through improved CT imaging. This prompts the recommendation of wedge resection and segmentectomy for early non-small cell lung cancer, yet the complexity of segmentectomy planning, relying on 3D reconstructions with on-screen limitations, remains a hurdle.

To tackle these challenges, Li et al. propose a new approach, combining 3D printing and augmented reality (AR) technology. Creating 3D-printed lung models for pre-surgery planning gives surgeons a better view, addressing issues seen with traditional on-screen models. This innovation extends to the operating room, where tangible models, brought to life with AR, significantly improve surgeons’ vision. The integration shows practical benefits, resulting in shorter surgery times, less blood loss, and shorter hospital stays.

The effectiveness of 3D printing and AR goes beyond surgery, aiding in the detailed task of identifying intersegmental planes during lung segmentectomy and reshaping surgical practices. Beyond immediate applications, these technologies offer promise for medical education and surgical training, providing a hands-on learning experience for students dealing with the complexities of lung structures.

Moving to collaborative insights, Chiou et al.’s (30) AR system and Sedeghi et al. (31) PulmoVR tool, combined with Chen et al.’s (32) contributions, paint a vivid picture of cutting-edge advancements in future surgical operations. Chiou et al.’s (33) AR system shines for its intuitive spatial information and cost-effectiveness, particularly useful in resource-limited settings. Similarly, Moawad et al.’s (34) exploration of AI-enhanced AR overlays, offered real-time support for surgeons providing data and guidance. This integration of AI augments the surgeon’s capabilities by offering intelligent support, such as identifying anatomical structures, providing diagnostic insights, or assisting in decision-making during surgery improving their precision and efficiency.

Addressing the complexities of pulmonary segmentectomies, the PulmoVR tool emerges as a potential player. This AI and VR-based planning tool navigates the challenges providing quick and comprehensive evaluations of patient-specific CT scans in immersive 3D. PulmoVR’s strengths, efficiency, cost-effectiveness, and user-friendly immersive features—signal a new era of realistic in-depth perception (31). This amalgamation of studies shows a future of a dynamic intersection where AI, AR, and VR converge to enhance surgical practices.

### 4.3 Real-time decision support

The intersection of diagnoses and treatments in lung tumor management is a critical area explored by Liu et al. (35) The study emphasizes the challenges faced by specialists in managing slowly increasing lesions, highlighting the necessity for rapid on-site accurate diagnoses for effective surgical strategies in early-stage non-small-cell lung cancer. The integration of Optical Coherence Tomography (OCT) with Artificial Intelligence (AI) stands out as a promising solution to address the current time lag in obtaining post-surgery definite diagnoses. OCT’s continuous slice images, particularly when integrated with AI (OCT-AI), demonstrate improved discrimination capabilities over traditional Frozen Sections (FS). Despite achieving an 80% accuracy rate, misclassifications are acknowledged, especially in scenarios with coexisting invasive and non-invasive features.

The study delves into the significance of tumor spread through air spaces (STAS) and OCT-AI’s potential to suggest wide excision for small tumors with invasive adenocarcinoma (IA) features. Convolutional Neural Networks (CNNs) are justified for AI training, with a focus on image classification in lung cancer. The study employs t-distributed stochastic neighbor embedding (t-SNE) for model visualization and gradient class activation mapping (grad-CAM) for evaluating salient features. The development of an interactive human–machine interface (HMI) is highlighted, offering clinicians real-time information and additional probability data for decision-making, even in cases of misclassifications. The study concludes by recognizing the OCT-AI system’s potential as an optional tool for rapid on-site diagnoses, with a commitment to continuous improvement and validation (35).

In parallel, Pao et al.’s (17) study illuminates the significant role of AI in enhancing intraoperative decision-making. DL algorithms are showcased for their ability to discern subtle pathological features intraoperatively, providing instantaneous insights into tissue histology. This real-time histopathological analysis facilitates dynamic adaptation of surgical approaches, ensuring thorough resection while minimizing unnecessary tissue excision. Thus, the integration of AI as a real-time decision support tool could enhance the intraoperative phase by combining surgeons’ expertise with AI’s analytical acumen, to bring about sophistication in lung cancer surgery.

The juxtaposition of these studies reveals a dichotomy between traditional surgical decision-making processes, influenced by factors like patient values, emotions, and decision complexity, as highlighted by Loftus et al. (6), and the potential advantages offered by AI-driven decision support systems. While the traditional model grapples with challenges and limitations, AI offers a transformative paradigm shift. Machine learning and deep learning present advantages in predicting medical outcomes, addressing the constraints of traditional approaches. Reinforcement learning further demonstrates AI’s versatility in optimizing specific clinical decisions. The synthesis of these studies marks a promising future in advancing the sophistication and efficacy of intraoperative decision-making in lung cancer surgery.

## 5 Postoperative period

### 5.1 Pathological assessment and margin evaluation

In the aftermath of lung cancer surgery, the postoperative period unfolds as a critical phase where AI demonstrates its potential in pathological assessment. A multitude of studies, such as those by Sheikh (36) and DiPalma et al. (37), accentuate the role of DL methodologies in detailed histological subtyping. These studies looked into the complexities of a 5-class problem, addressing various histological patterns encompassing lepidic, acinar, papillary, micropapillary, and solid patterns. Sheikh et al. (36) explored the impact of multiple descriptors on a deep learning model’s performance in the multi-class classification of WSIs. They found that augmenting inputs enhanced the discriminatory capabilities of the model. DiPalma et al. (37) introduced a Knowledge Distillation (KD) method, showcasing its superiority over baseline metrics in effectively classifying diseases such as celiac disease and lung adenocarcinoma. These studies highlight how AI not only aids in subtyping histology but also contributes to a more nuanced understanding of lung cancer, providing clinicians with a comprehensive diagnostic toolkit.

The emphasis on accurate subtyping is particularly pertinent in lung cancer, given its heterogeneity and the subsequent challenges it poses to precise pathological interpretation. However, a critical examination beckons: to what extent can AI truly replicate the expertise of pathologists in discerning these intricate patterns?

Moreover, the postoperative period demands a meticulous evaluation of surgical margins. The study by Kanavati et al. (7) delves into the prowess of DL algorithms in real-time surgical guidance, significantly impacting margin assessment. It raises an intriguing proposition — can AI serve as an adjunct, offering a second layer of scrutiny to ensure optimal margins? The challenge lies not only in the technical accuracy of AI but also in establishing a seamless integration with existing pathological workflows.

### 5.2 Prognostic insights and predictive modelling

The landscape of postoperative medical research unfolds with the synergy of artificial intelligence (AI) in treatment prediction. Dercle et al.’s (38) AI model represents a significant stride in predicting therapy responses, contributing to nuanced treatment decision-making. Simultaneously, concerns arise in ANN studies, notably regarding overfitting due to oversized networks, prompting reflections on reliability and validation. Ongoing efforts address these challenges, exemplified by a novel ANN tool tailored for small datasets and the underexplored potential of ensemble runs. The importance of refined variable selection comes to the forefront, with pruning emerging as a method to enhance input-to-output relationships in ANNs. Acknowledging the advantages of ANNs, such as learning without prior knowledge and suitability for clinical tasks, contrasts with persistent issues of overfitting. Within this context, the dynamic approach of ANNs in analyzing mortality risk, accommodating outliers and nonlinear interactions, underscores their potential amid challenges (39–43).

Shifting focus to lung cancer prognosis, AI plays a pivotal role in addressing multifaceted factors influencing outcomes, including age, tumor characteristics, and treatment modalities (44). Recognizing the limitations of single-test item prognostication, studies advocate for AI-integrated predictive models to enhance accuracy (44). Recent research illustrates the application of deep learning and imaging analyses, such as PET, effectively staging lung cancer (45). Notably, eXtreme Gradient Boosting (XGBoost), a deep learning library, contributes to constructing models by sorting feature importance based on decision tree models (46). Utilizing this approach, predictive models like the ITEN model offer personalized drug treatment recommendations, particularly for cases with bone metastasis, ultimately improving patient survival rates. The evolution of AI-driven models, incorporating neural networks in deep learning, signals a paradigm shift in treatment optimization. The ITEN model’s consistency with published data reaffirms its reliability in predicting survival efficiency in non-small cell lung cancer patients (46). Integrating these advancements with ongoing efforts in AI-driven treatment predictions and evolving ANN methodologies enriches the landscape of medical research, providing a comprehensive approach to lung cancer prognosis and treatment optimization.

As the postoperative period sounds like a promising field for AI integration into routine clinical workflows, the concomitant challenges cannot be overlooked. The integration of digital pathology, as advocated by Pao et al. (17), necessitates a paradigm shift in infrastructure, storage, and data-sharing practices. The ethical dimension becomes pronounced, with concerns regarding patient data privacy and the development of regulatory frameworks surfacing prominently. Moreover, the dependence on extensive labeled datasets for training raises questions about the representativeness of these datasets and the potential biases embedded within them.

while AI promises to revolutionize the postoperative management of lung cancer, its implementation requires a judicious approach. The balance must be meticulously struck to ensure optimal outcomes in the postoperative care continuum (4).

## 6 Challenges and limitations

The integration of artificial intelligence (AI) into lung cancer management, as elucidated in the reviewed studies, brings forth a spectrum of challenges and limitations that merit careful consideration.

### 6.1 Lack of interpretability

A salient concern extracted from the systematic review is the conspicuous lack of interpretability and explanation within certain AI applications (47). While the proficiency of AI models in classification tasks is evident, the paucity of concerted efforts in addressing common sense reasoning, especially in deciphering the intricate physical characteristics of cells, poses a critical challenge (36). This interpretability gap, particularly pronounced in tasks requiring nuanced understanding, introduces a layer of complexity in the integration of AI insights into the clinical decision-making process. As clinicians often rely on interpretive skills honed through years of training, the opaque nature of AI outputs may impede the establishment of trust and hinder widespread adoption.

### 6.2 Training limitations with inadequate samples

The depth of learning algorithms, highlighted by multiple studies (8, 9, 36, 39, 41–43) necessitates substantial volumes of labeled data for effective performance. However, the pragmatic challenge arises when dealing with the sheer scale of annotations required, often dependent on the expertise of pathologists (37). This challenge is exacerbated in scenarios where specific histopathological subtypes or rare molecular profiles are encountered infrequently. The resultant scarcity of comprehensive datasets compromises the generalizability of AI models. Addressing this challenge demands collaborative initiatives for the meticulous curation of diverse datasets that mirror the true heterogeneity encountered in clinical practice.

### 6.3 Less power in problems beyond classification

While the prowess of AI, especially deep learning, in classification tasks is evident, the studies underscore its relatively diminished efficacy in addressing problems beyond classification (3). In the expansive landscape of lung cancer management, where intricate analyses involving regression, clustering, and multi-dimensional correlations are often required, the limitations of AI become apparent. Traditional machine learning techniques, capable of handling diverse problem sets, might outshine deep learning in these nuanced domains (48). This prompts a critical reflection on the strategic deployment of AI, emphasizing its alignment with the specific analytical demands of the clinical context.

### 6.4 Lack of global generalization

The pervasive challenge of the lack of global generalization in deep learning algorithms emerges consistently across studies (16, 26, 27, 49, 50). Overfitting tendencies, wherein models excel in performance on training data but falter when presented with new or unlabeled data, pose a substantial challenge. In the context of lung cancer diagnosis, characterized by variations in imaging techniques and equipment, achieving robust generalization becomes a formidable task (47). The demand for models that can seamlessly adapt to diverse clinical settings is not only an academic concern but a practical necessity for the broader implementation of AI in lung cancer care.

However, challenges to the widespread adoption of AI in healthcare are acknowledged, encompassing issues of data standardization, technology infrastructure, interpretability, safety, monitoring, and ethical considerations (19). The need for rigorous analysis, external validation, and mitigation of biases in training data is emphasized, particularly given the potential consequences of algorithmic errors (19). The ethical challenges surrounding biases in algorithm outputs and accountability underscore the necessity for a robust regulatory framework for AI in healthcare (19).

### 6.5 High memory and computational cost requirements

The ambition to deploy deep learning models in lung cancer diagnosis is tempered by the pragmatic constraints of high memory and computational costs, as underscored by (3, 51). The intricate nature of biopsy images, often high in resolution, demands sophisticated processing capabilities. This raises pertinent questions about the scalability and accessibility of such approaches in real-world healthcare settings. While advancements are underway to optimize computational efficiency, the inherent resource demands remain a critical consideration in the practical implementation of AI in routine clinical workflows.

In summary, the journey of AI in lung cancer management is not devoid of hurdles. A critical understanding of these challenges, fortified by insights from the review, becomes imperative for steering the trajectory of AI research and application toward meaningful and sustainable integration into lung cancer care.

### 6.6 Ethical dilemmas

The integration of Artificial Intelligence (AI) in thoracic surgery, especially in lung cancer treatment, brings to the forefront a spectrum of ethical considerations that are critical for maintaining patient welfare and integrity in medical practice. Central to this ethical framework is ensuring patient autonomy through informed consent, as AI’s involvement in diagnostic and surgical decision-making introduces complexities requiring patient comprehension of AI’s influence on treatment options and conscious choice-making (52). This aligns with the imperative need to safeguard patient data privacy and security, addressing the ethical challenges posed by AI’s reliance on extensive health data for operation, thereby keeping patient trust and confidentiality (53). Equally crucial is addressing potential biases in AI, given its dependency on training data, to prevent the perpetuation of healthcare disparities, particularly in lung cancer treatment where demographic differences are significant (53, 54). Furthermore, the opacity of AI systems necessitates a robust approach to transparency and accountability, ensuring that AI supplements rather than supplants the expert clinical judgment of healthcare professionals (54, 55). The ethical integration of AI in thoracic surgery demands continuous monitoring and evaluation to assess its accuracy, effectiveness, safety, and overall impact on patient outcomes, ensuring that AI’s deployment remains aligned with ethical standards and patient-centric values.

## 7 Conclusion

In conclusion, while the integration of artificial intelligence (AI) in lung cancer management shows promise across phases, it faces notable challenges. In the preoperative phase, AI enhances diagnostics and predicts molecular biomarkers, especially in cases with limited biopsy materials. Intraoperatively, AI transforms surgery by providing real-time guidance and decision support. Postoperatively, AI aids in pathological assessment and predictive modeling for refined care.

However, challenges include the lack of interpretability, training limitations affecting model generalizability, and AI’s efficacy beyond classification. Global generalization, marked by overfitting, poses a challenge, along with high memory and computational costs and challenging ethical frameworks. Addressing these challenges requires a judicious approach, considering ethical, technical, and regulatory dimensions. Rigorous analysis, external validation, and a robust regulatory framework are crucial for responsible AI implementation in lung cancer care, emphasizing the evolving intersection of human expertise and technological advancement.

## Author contributions

NA: Conceptualization, Writing – original draft, Writing – review & editing. FM: Writing – review & editing. AG: Writing – review & editing. PS: Writing – review & editing. UC: Writing – review & editing. MS: Conceptualization, Writing – review & editing.
